# Role of the Pseudomonas plecoglossicida fliL gene in immune response of infected hybrid groupers (Epinephelus fuscoguttatus ♀ × Epinephelus lanceolatus ♂)

**DOI:** 10.3389/fimmu.2024.1415744

**Published:** 2024-07-04

**Authors:** Lian Shi, Lingmin Zhao, Qi Li, Lixing Huang, Yingxue Qin, Zhixia Zhuang, Xiaoru Wang, Huabin Huang, Jiaonan Zhang, Jiaolin Zhang, Qingpi Yan

**Affiliations:** ^1^ Fisheries College, Jimei University, Xiamen, China; ^2^ College of Environment and Public Health, Xiamen Huaxia University, Xiamen, China; ^3^ Key Laboratory of Special Aquatic Feed for Fujian, Fujian Tianma Technology Company Limited, Fuzhou, China

**Keywords:** *Pseudomonas plecoglossicida*, *FliL*, pathogenicity, inflammation, immune response, hybrid grouper

## Abstract

*Pseudomonas plecoglossicida*, a gram-negative bacterium, is the main pathogen of visceral white-point disease in marine fish, responsible for substantial economic losses in the aquaculture industry. The FliL protein, involved in torque production of the bacterial flagella motor, is essential for the pathogenicity of a variety of bacteria. In the current study, the *fliL* gene deletion strain (Δ*fliL*), *fliL* gene complement strain (C-Δ*fliL*), and wild-type strain (NZBD9) were compared to explore the influence of the *fliL* gene on *P. plecoglossicida* pathogenicity and its role in host immune response. Results showed that *fliL* gene deletion increased the survival rate (50%) and reduced white spot disease progression in the hybrid groupers. Moreover, compared to the NZBD9 strain, the Δ*fliL* strain was consistently associated with lower bacterial loads in the grouper spleen, head kidney, liver, and intestine, coupled with reduced tissue damage. Transcriptomic analysis identified 2 238 differentially expressed genes (DEGs) in the spleens of fish infected with the Δ*fliL* strain compared to the NZBD9 strain. Based on Kyoto Encyclopedia of Genes and Genomes (KEGG) enrichment analysis, the DEGs were significantly enriched in seven immune system-associated pathways and three signaling molecule and interaction pathways. Upon infection with the Δ*fliL* strain, the toll-like receptor (TLR) signaling pathway was activated in the hybrid groupers, leading to the activation of transcription factors (NF-κB and AP1) and cytokines. The expression levels of proinflammatory cytokine-related genes *IL-1β*, *IL-12B*, and *IL-6* and chemokine-related genes *CXCL9*, *CXCL10*, and *CCL4* were significantly up-regulated. In conclusion, the *fliL* gene markedly influenced the pathogenicity of *P. plecoglossicida* infection in the hybrid groupers. Notably, deletion of *fliL* gene in *P. plecoglossicida* induced a robust immune response in the groupers, promoting defense against and elimination of pathogens via an inflammatory response involving multiple cytokines.

## 1 Introduction

Bacterial diseases, induced by various factors, continue to represent a considerable challenge within the aquaculture industry (1–4). *Pseudomonas plecoglossicida*, a conditionally pathogenic bacterium with a polar flagellum, causes a wide range of fish diseases, predominantly under low-temperature conditions (5–8). Recent reports have underscored the high infectivity and mortality rates of visceral white spot disease in large yellow croakers (*Larimichthys crocea*) and orange-spotted groupers (*Epinephelus coioides*) (6, 9). The pathogenic mechanisms employed by bacterial pathogens in fish involve various virulence factors, including toxic extracellular products (enzymes and toxins), biofilms, virulence genes, and mobile genetic factors for antimicrobial resistance (1, 10, 11). Genes such as *pvdE*, *rpoD*, and *znuC* are closely related to the virulence regulation of *P. plecoglossicida* and play critical roles in the host infection process (12–14). In addition, dual RNA sequencing (RNA-seq) analyses previously performed in our laboratory on the spleens of orange-spotted groupers infected with *P. plecoglossicida* revealed up-regulation of the *fliL* gene in the host, suggesting its potential involvement in the regulation of *P. plecoglossicida* virulence.

Flagella serve as essential motile organs in bacteria, playing a pivotal role in bacterial adhesion and invasion (15). The flagellar matrix, consisting of a rotor and multiple surrounding stator units, operates as a rotating motor that generates torque to turn the flagellar filament, facilitating bacterial movement (16). FliL, a membrane protein primarily localized in the periplasm, usually forms a ring around each stator unit, necessary for stator activation (17). In *P. aeruginosa*, FliL acts in synergy with the MotAB stator to maintain a high motor switching rate and with the MotCD stator to increase motor speeds (18). While *fliL* gene deletion does not affect the cellular morphology or flagella of *Vibrio alginolyticus*, it does significantly reduce swimming speed, especially at higher loads (16). In addition, FliL is associated with the swimming or adhesion mechanisms of *Clostridium difficile*, and deletion of *fliL* affects the ability of cells to sense surfaces (19). To date, however, the effects of *fliL* on *P. plecoglossicida* and its pathogenicity remain unexplored.

Therefore, in the current study, we compared bacterial load and tissue damage in the spleen, head kidney, liver, and intestine of hybrid groupers infected with the *fliL* gene deletion strain (Δ*fliL*) and wild-type strain (NZBD9) to explore the influence of *fliL* on *P. plecoglossicida* pathogenicity. Differences in splenic immune responses of hybrid groupers infected with the Δ*fliL* or NZBD9 strain were analyzed by RNA-seq to reveal the role of *fliL* in the *P. plecoglossicida* infection process within the host.

## 2 Materials and methods

### 2.1 Bacterial strains and culture conditions

The wild-type strain of *P. plecoglossicida* (NZBD9) used in this study was previously isolated from large yellow croaker with visceral white spot disease in our laboratory (20). The Δ*fliL* strain was obtained by homologous recombination after knocking out *fliL* gene of wild strain, and then replacing *fliL* gene to obtain C-Δ*fliL* strain (21). Bacteria were cultured in Luria-Bertani (LB) broth containing ampicillin (100 µg/mL) or tetracycline (10 µg/mL) at 18 °C and 220 rpm.

### 2.2 Experimental fish and feeding

Approximately 50 g of healthy hybrid grouper with body surface free of wounds and parasites were purchased from a Xiamen farm (Fujian, China) and temporarily cultured in a circulating mariculture system for two weeks to adapt to the experimental conditions. The temperature was maintained at 18 ± 1 °C, with commercial feed of no more than 3% of body weight provided daily during the temporary rearing period.

### 2.3 Ethics statement

All animal experiments were approved by the Ethics Committee of Jimei University (license No. JMULAC201159) and were conducted in accordance with the Guidelines for the Care and Use of Laboratory Animals of the National Institutes of Health.

### 2.4 Artificial infection experiment

#### 2.4.1 Preparation of bacterial suspension

The NZBD9, Δ*fliL*, and C-Δ*fliL* strains of *P. plecoglossicida* were cultured in LB broth at 18 °C with shaking at 220 rpm overnight. After centrifugation at 4 °C and 4 000 rpm for 10 min, the bacteria were collected and washed with phosphate-buffered saline (PBS) to prepare a bacterial suspension at a dose of 5 × 10^4^ CFU/fish. Sterile PBS was prepared for injection into the control group.

#### 2.4.2 Mortality measurement

A total of 120 hybrid groupers of similar size and specifications were randomly divided into four groups containing 30 groupers each. The fish were inoculated with a 200-μL injection of the NZBD9 strain, Δ*fliL* strain, C-Δ*fliL* strain, or sterile PBS (negative control) into the tail. Observations were made every 12 h after the injection to record the number of fish that died each day. The dead fish were dissected to observe their internal organs and take photographs. Continuous observations and recordings were performed for 10 days.

#### 2.4.3 Tissue sampling

A total of 240 fish were randomly divided into a NZBD9 strain-injected group, Δ*fliL* strain-injected group, and PBS-injected group, with the injection dose and method as described above. The spleen, liver, head kidney, and intestine were collected at 4 days post-injection (dpi) and stored in 50 mL of fixed solution (37.5 mL of 4% paraformaldehyde, 10 mL of ethanol, 2.5 mL of ice acetic acid) for histopathological observations. The same organs were collected from three fish in each group at 1–6 dpi for bacterial load determination. Additionally, spleens were collected from three fish in each group at 0–6 dpi to detect the expression of host immune-related genes, while the spleens of three fish from each group were collected at 4 dpi for transcriptome sequencing. All samples were temporarily placed in liquid nitrogen before transfer to −80 °C for storage.

### 2.5 Histopathological sections

Tissue samples were fixed for more than 24 h, then removed and gently rinsed 2–3 times with ultrapure water. After gradient dehydration in 70%–100% ethanol (20 min each), the samples were successively transferred to ethanol/xylene (1:1) mixture and xylene solution (twice) for transparency (20 min each), infiltrated in paraffin for 1 h at 65 °C, then embedded in paraffin. After coagulation, the tissue samples were cut into 7-μm slices in a microtome and spread on a glass slide with distilled water and dried at 37 °C.

A hematoxylin and eosin (H&E) staining kit (C0105S, Beyotime, Shanghai, China) was used according to the manufacturer’s protocols. The slides were dewaxed in xylene solution twice (10 min each), rehydrated with 100%–70% ethanol (2 min each), stained with hematoxylin solution for 5–10 min, and rinsed with running water for 10 min. The slides were then differentiated with hydrochloric acid ethanol solution for 15 s, rinsed with tap water, and stained with eosin solution for 1 min, with excess dye subsequently rinsed off. The slides were then dehydrated with 70%–100% gradient ethanol (10 s each), transparentized in xylene twice (5 min each), sealed with neutral gum and scanned under a Leica microscope after drying.

### 2.6 Bacterial load measurement

The DNA of each tissue sample from the infected hybrid groupers was extracted using an EasyPure Marine Animal Genomic DNA kit (TransGen Biotech, China) according to the manufacturer’s instructions. Copy number of the *P. plecoglossicida* housekeeper gene *gyrB* was used to measure bacterial load in each spleen sample (22). Analysis was performed by quantitative real-time polymerase chain reaction (qRT-PCR), performed using the QuantStudio 6 Flex Real-Time Fluorescent Quantitative PCR system (Life Technologies, USA). The reaction system (10 μL) was: 5 μL of 2 × PerfectStart Green qPCR SuperMix (TransGen Biotech, China), 0.4 μL of 10 μM upper and downstream primers, 0.5 μL of template DNA, and 3.7 μL of nuclease-free water. The *gyrB* primer sequence is shown in **Supplementary Table 1**.

### 2.7 Quantitative analysis of immune-related gene expression

Total RNA of each spleen sample from the infected hybrid groupers was extracted using the TransZolUpKit (TransGen Biotech, China) according to the manufacturer’s instructions. The cDNA was synthesized using TransScript All-in-One First-Strand cDNA Synthesis SuperMix for qRT-PCR (One-Step gDNA Removal) (TransGen Biotech, China). The *β-actin* gene was used as a reference gene for qRT-PCR analysis, and the relative mRNA expression of the target gene was calculated using the 2^-ΔΔCT^ method (23). The reaction system was the same as above. The relevant genetic primers are listed in **Supplementary Table 1**.

### 2.8 RNA-seq and analysis

To explore the effects of *fliL* from *P. plecoglossicida* on the immune response of the hybrid groupers, non-reference transcriptomic analysis was carried out on the spleens collected from the NZBD9 and Δ*fliL* strain-injection groups at 4 dpi. Total RNA was extracted from each spleen sample (30 mg) as described above. RNA concentration and purity were detected using a NanoDrop 2000, and RNA integrity was detected by agarose gel electrophoresis. The RNA integrity number (RIN) was determined using an Agilent 2100 Bioanalyzer. A Ribo-Zero rRNA kit (Epicentre, USA) was used to remove ribosomal RNA (rRNA). An Illumina TruSeq™ RNA Sample Prep kit was used to construct a library. Sequencing was performed on the Illumina NovaSeq 6000 platform (read length 2 × 150 bp) at Majorbio Bio-Pharm Technology Co., Ltd. (Shanghai, China).

Fastp software was used to conduct quality control of the original sequencing data, with high-quality (clean) data obtained to ensure the accuracy of subsequent analyses (24). Trinity was used to assemble all clean data (25). TransRate and CD-HIT were used to optimize and filter the obtained initial assembly sequence and BUSCO was used to perform assembly evaluation (26–28). The clean reads of each sample were finally aligned with the reference sequences obtained by Trinity assembly, and the mapped reads of each sample, which could be aligned to the number of clean reads on the assembly transcript, were obtained for subsequent quantitative analysis of genes and transcripts.

Gene and transcript expression levels were quantified using RSEM with transcripts per million (TPM) as the metric of expression (29, 30). DESeq2 was used to identify differentially expressed genes (DEGs) between groups, applying a significance threshold of *P*-adjusted < 0.05 and |log_2_ FC| ≥ 1 (31). Gene Ontology (GO) and Kyoto Encyclopedia of Genes and Genomes (KEGG) functional enrichment analyses were performed using GOATOOLS and KOBAS, respectively (32, 33). The Fisher method was used for accurate test, and after multiple test correction based on BH method, FDR < 0.05 indicated that there was significant enrichment of this function. In addition, eight DEGs were randomly selected from the transcriptomic analysis results, and the reliability of the RNA-seq results was tested by qRT-PCR. Details on software and validation primers used in this process are provided in **Supplementary Tables 2** and **3**, respectively.

### 2.9 Drawing and statistical analysis

GraphPad Prism (v9.0.0) and Adobe Illustrator (USA) were used for drawing and image typesetting. All data are expressed as mean ± standard deviation (SD). IBM SPSS Statistics v26.0 (USA) was used to conduct one-way analysis of variance (ANOVA) and Dunnett’s multiple comparison tests, with *P* < 0.05 considered statistically significant.

### 2.10 Data access

The RNA-seq data were deposited in the NCBI GenBank SRA database under accession numbers SRP432909 (NZBD9 strain group) and SRP433934 (Δ*fliL* strain group).

## 3 Results

### 3.1 Effects of *fliL* on *P. plecoglossicida* pathogenicity

**Figure 1:**
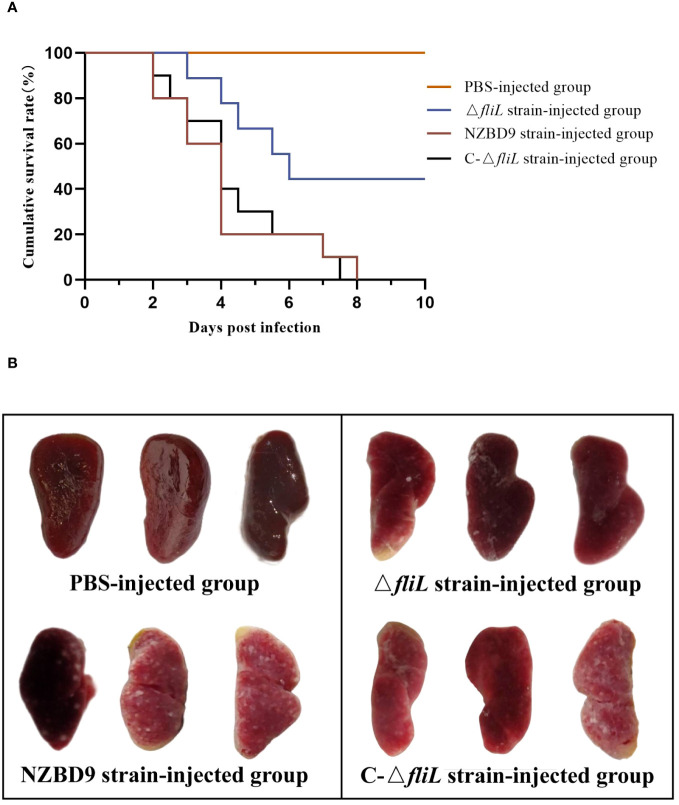
Pathogenicity of *P. plecoglossicida* NZBD9, Δ*fliL*, and C-Δ*fliL* strains to hybrid groupers. **(A)** Cumulative survival curve of each injection group of hybrid groupers; **(B)** Spleen of each injection group of hybrid groupers.

The 10-day cumulative survival rate of hybrid groupers artificially infected with the *P. plecoglossicida* NZBD9, Δ*fliL*, or C-Δ*fliL* strains is shown in **Figure 1A**. Fish injected with the NZBD9 or C-Δ*fliL* strains began to die at 2 dpi, with the survival rates decreasing to 0% by 8 dpi and 7.5 dpi, respectively. Conversely, mortality among fish infected with the Δ*fliL* strain occurred one day later than those injected with the NZBD9 and C-Δ*fliL* strains, and no further deaths were observed after 6 dpi, resulting in a final survival rate of 50%. The PBS-injected control group exhibited no mortality. During the later stages of infection (4–6 dpi), white nodules appeared on the spleens of infected hybrid groupers. **Figure 1B** shows the spleen anatomy of fish from each injection group at 4 dpi, revealing numerous white nodules on the surface of the spleen in fish infected with the NZBD9 or C-Δ*fliL* strains, while the spleens of the Δ*fliL*-infected fish contained only a few white spots and the spleens of the PBS-injected fish showed a smooth surface and no white spots. These findings indicate that the virulence of the Δ*fliL* strain was weaker than that of the NZBD9 and C-Δ*fliL* strains, highlighting the significant impact of *fliL* on *P. plecoglossicida* pathogenicity.

### 3.2 Bacterial load in tissues of hybrid groupers infected with NZBD9 and Δ*fliL* strains

**Figure 2:**
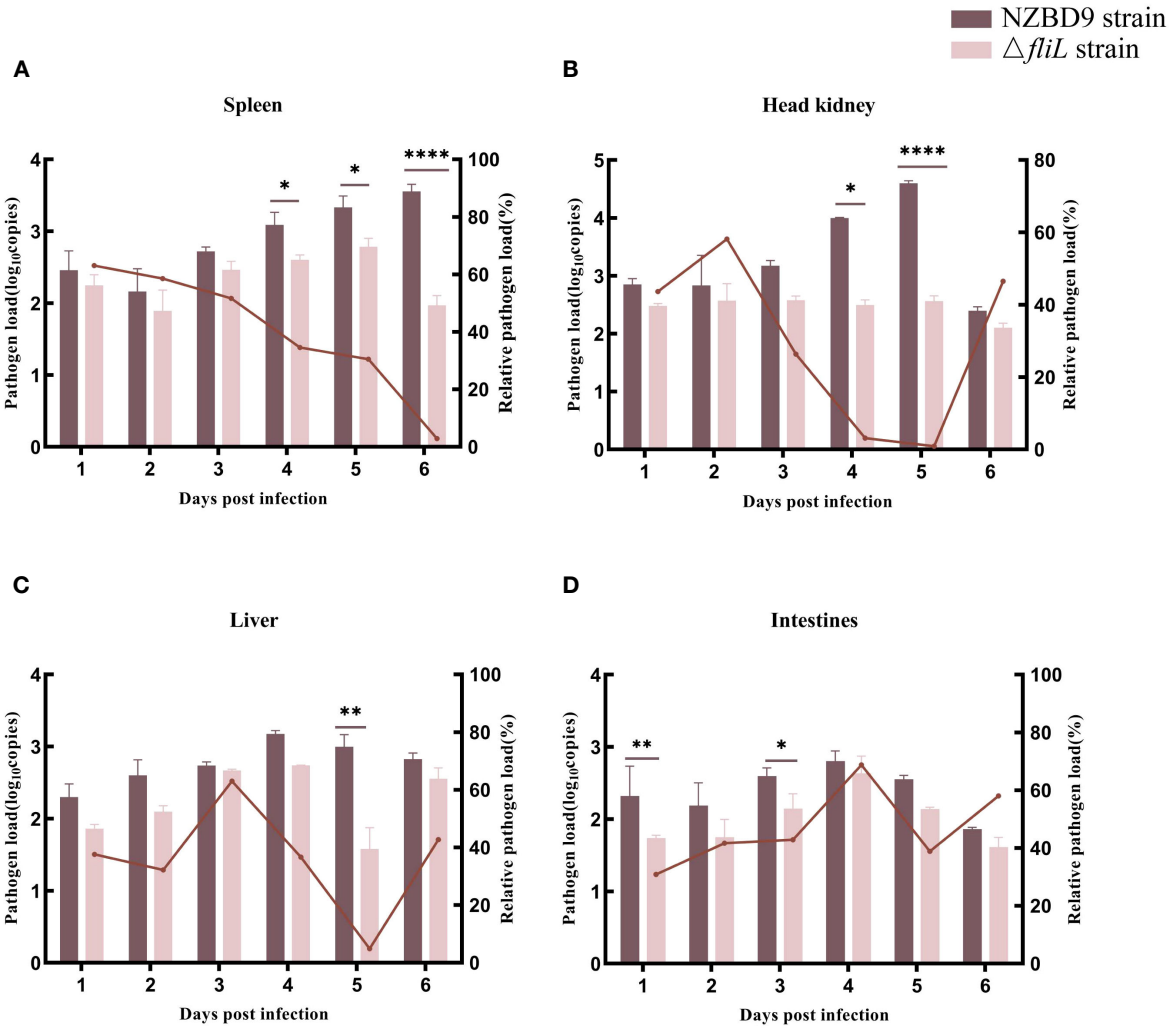
Bacterial loads of NZBD9 and Δ*fliL* strains in hybrid grouper tissues during infection. Column chart shows pathogen load, represented by *gyrB* gene copy number. Line plots indicate relative pathogen load (*gyrB* gene copy number in grouper tissue infected with Δ*fliL* strain/*gyrB* gene copy number in grouper tissue infected with NZBD9 strain). **(A)** Spleen. **(B)** Head kidney. **(C)** Liver. **(D)** Intestine. All data are expressed as mean ± SD, n = 3. Differences between two groups are indicated by asterisks. ^*^ *P* < 0.05, ^**^ *P* < 0.01, ^****^ *P* < 0.0001.

The bacterial load of the Δ*fliL* strain was lower than that of the NZBD9 strain in the spleen, head kidney, liver, and intestine of hybrid groupers from 1 to 6 dpi. As infection time progressed, the bacterial load of the NZBD9 strain increased in the spleen and head kidney. The bacterial load of the Δ*fliL* strain in the spleen peaked at 5 dpi, differing significantly (*P* < 0.05) from the NZBD9 strain in the later infection stages (4–6 dpi) (**Figure 2A**). The bacterial load of the Δ*fliL* strain in the head kidney remained relatively stable throughout the infection period (**Figure 2B**). The bacterial loads of both the NZBD9 and Δ*fliL* strains showed an increasing then decreasing trend in the liver and intestine, with significant differences (*P* < 0.05) observed at 1 dpi, 3 dpi, and 5 dpi, respectively (**Figures 2C, D**).

### 3.3 Histopathological changes in infected groupers

**Figure 3:**
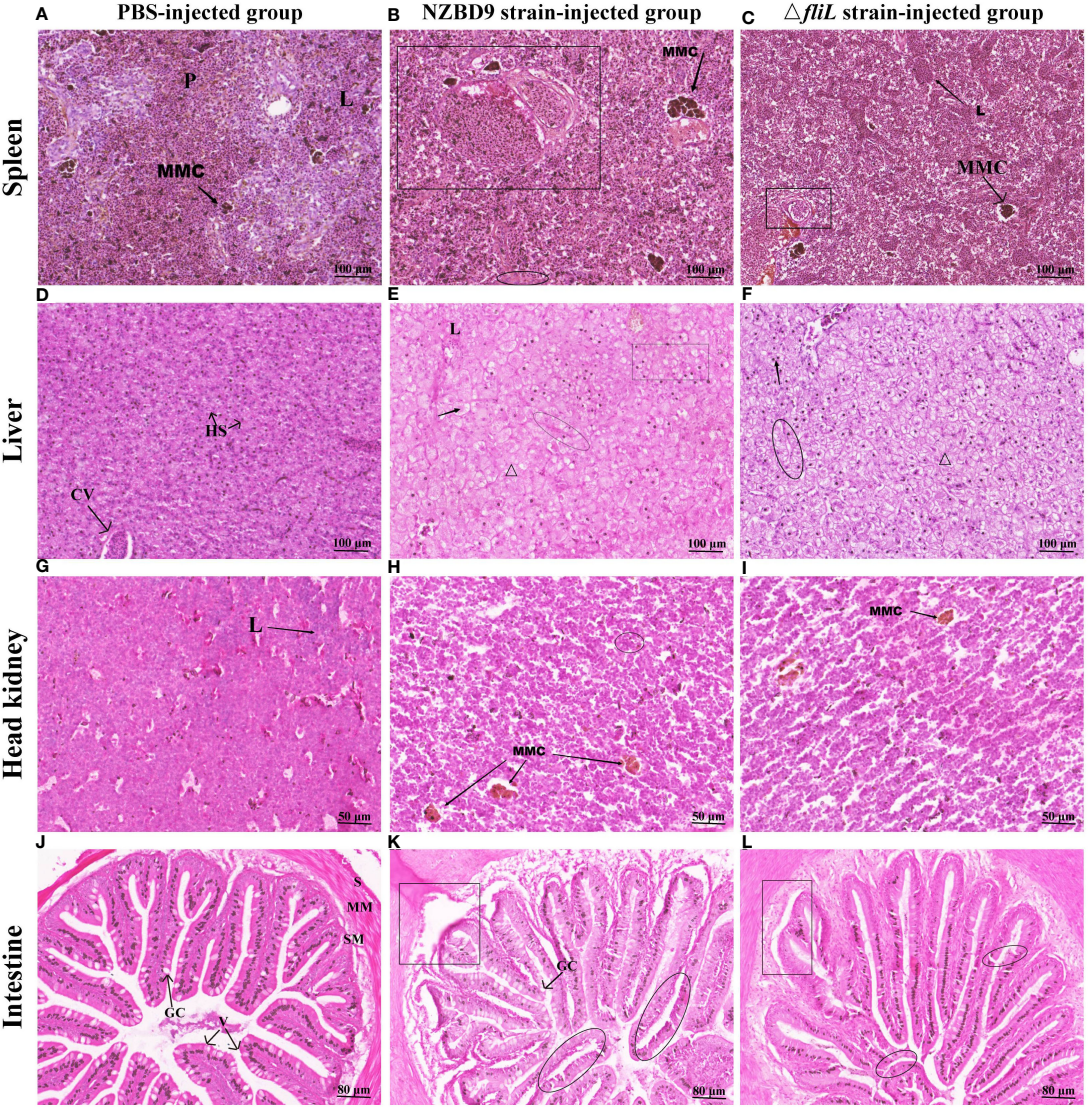
Histopathological changes in hybrid groupers infected with NZBD9 or Δ*fliL* strains. Spleen: **(A)** PBS-injected group. Red and white medulla (P), melanin macrophage center (MMC), lymphocytes (L); **(B)** NZBD9 strain-infected group. Granulomatous tissue (◻), hemolysis and necrosis of tissue (○); **(C)** Δ*fliL*-infected group. Lymphocyte aggregation (↖); Liver: **(D)** PBS-injected group. Hepatic blood sinusoids (HS), central vein (CV); **(E)** NZBD9-infected group. Loss of nuclei (↗), disorganized cell arrangement (Δ), hemocyte agglutination (○), vacuolization (▭); **(F)** Δ*fliL*-infected group. Nuclear loss (Δ), deviated nuclei (↖), blurred cell boundaries (○); Head kidney: **(G)** PBS-injected group; **(H)** NZBD9-infected group. MMC (↙), hemocyte agglutination (○); **(I)** Δ*fliL*-infected group. MMC (↘); Intestine: **(J)** PBS-injected group. Plasma membrane layer (S), mucosal muscular layer (MM), submucosal layer (SM), villi (V), goblet cells (GC); **(K)** NZBD9-infected group. Shedding of intestinal villi (◻), internal division of villi (○); **(L)** Δ*fliL*-infected group. Separation of muscularis mucosae from mucosal layer (▯), slight breakage of villi (○).

H&E staining was performed on the spleen, liver, head kidney, and intestine of groupers exposed to either the NZBD9 or Δ*fliL* strains to compare changes in histopathology. In the PBS-injected group, normal splenic tissues exhibited a mixed distribution of red and white medulla, with a small number of melanin macrophage centers (MMCs) and lymphocytes (**Figure 3A**). In comparison, spleens from NZBD9-infected fish showed pronounced granulomatous tissue changes, with increased MMC area and evidence of tissue hemolysis and necrosis (**Figure 3B**). Conversely, Δ*fliL*-infected fish presented with reduced splenic granuloma sizes and MMC areas, alongside lymphocyte aggregates within the tissue (**Figure 3C**).

In liver samples from the PBS-injected group, hepatocytes were neatly arranged in a tightly packed formation, and the hepatic blood sinusoids and the central vein clearly visible (**Figure 3D**). In contrast, the NZBD9 and Δ*fliL*-infected groups (**Figures 3E, F**) exhibited disorganized cells with indistinct borders and occasional blood cell agglutination. These samples also displayed pathological features such as nuclear deviation, nuclear loss, and vacuolization, with these symptoms more pronounced in the NZBD9-infected fish. Compared to the PBS-injected group (**Figure 3G**), head kidney tissues in the NZBD9 and Δ*fliL*-injected groups showed dispersed structures and varying levels of MMC or hemocyte agglutination (**Figures 3H, I**). For the intestinal tissues, the PBS-injected group displayed preserved structures, including the serosal layer, mucosal muscle layer, submucosa, and well-formed villi—comprising mucosal epithelium and lamina propria extending into the lumen—with goblet cells distinctly visible (**Figure 3J**). In contrast, the NZBD9-injected group showed notable detachment and segmentation of intestinal villi, as well as a significant reduction in goblet cells (**Figure 3K**), while the Δ*fliL*-infected group showed separation between the muscle and mucosal layers and slight fracture of villi (**Figure 3L**).

### 3.4 Analysis of RNA-seq data from hybrid grouper spleen

Transcriptomic analysis was performed on six spleen samples, yielding a total of 48.59 Gb of clean data, including 6.42 Gb of clean data per sample. Quality metrics revealed a Q30 score of above 93.53% (percentage of total bases with sequencing quality of 99.9% or higher), with a G/C content of approximately 48% and an average error rate of sequenced bases < 0.1% (**Supplementary Table 4**). In addition, the distribution of A/T/G/C base content was uniform, with N% close to 0 (**Supplementary Figure 1**). Analysis identified a total of 77 567 unigenes and 111 098 transcripts across all samples. These findings indicate that the sequencing data were of good quality, suitable for further analysis.

**Figure 4:**
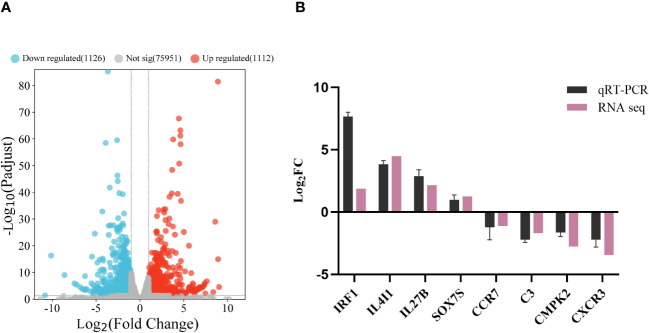
Analysis and validation of DEGs in the transcriptomes. **(A)** Volcano map of transcriptome gene expression. **(B)** qRT-PCR validation of transcriptome. Horizontal coordinate is the gene name, vertical coordinate is the multiple of expression difference.

Analysis identified 2 238 DEGs between the Δ*fliL* and NZBD9 strain transcriptomes, including 1 112 up-regulated and 1 126 down-regulated genes (**Figure 4A**). Four up-regulated and down-regulated DEGs were randomly selected for qRT-PCR detection. The gene expression trends from qRT-PCR were consistent with the sequencing results (**Figure 4B**). The Pearson correlation coefficient was greater than 0.8, indicating a significant correlation between the two (*P* < 0.05) (**Supplementary Figure 2**), confirming the reliability of the transcriptome data.

**Figure 5:**
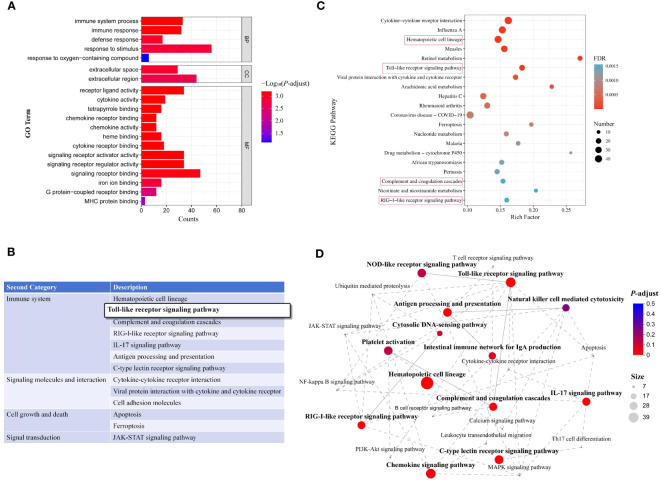
Enrichment analysis of DEGs in transcriptomes. **(A)** GO pathway enrichment analysis of DEGs (top 20); **(B)** Secondary classification of KEGG-enriched pathways; **(C)** Top 20 enriched KEGG pathways. Red rectangles represent immune system-related pathways. **(D)** Interactions among enriched immune system-related pathways.

GO enrichment analysis of DEGs was performed regarding enrichment in molecular function (MF), cellular component (CC), and biological process (BP) terms. Results showed that the DEGs were enriched in 160 GO secondary pathways, including 19 significantly enriched pathways. **Figure 5A** shows the top 20 enriched GO pathways, including many immune response-related pathways such as immune system processes, immune response, defense response, response to stimuli, receptor ligand activity, signal receptor regulatory activity, cytokine receptor binding, chemokine activity, cytokine activity, and signal receptor activator activity.

KEGG analysis indicated that the DEGs were enriched in 316 metabolic pathways, including 50 significantly enriched pathways (*P* < 0.05). Secondary KEGG categories included signaling molecules and interaction, immune system, immune disease, lipid metabolism, cell growth and death, bacterial infectious diseases, nucleotide metabolism, transport and catabolism, signal transduction, endocrine and metabolic disease, carbohydrate metabolism, and digestive system. Among the significantly enriched pathways, seven were associated with the immune system and three with signaling molecules and interactions (**Figure 5B**). The top 20 enriched KEGG pathways are shown in **Figure 5C**, including hematopoietic cell profiles, Toll-like receptor (TLR) signaling pathway, complement and coagulation cascade response, RIG-I-like receptor signaling pathway, and other immune system-related pathways. In addition, enrichment network analysis was performed for the top 13 immune system-related KEGG pathways (**Figure 5D**). Overall, these findings suggest that Δ*fliL* strain infection elicits a range of immune responses in hybrid groupers.

**Figure 6:**
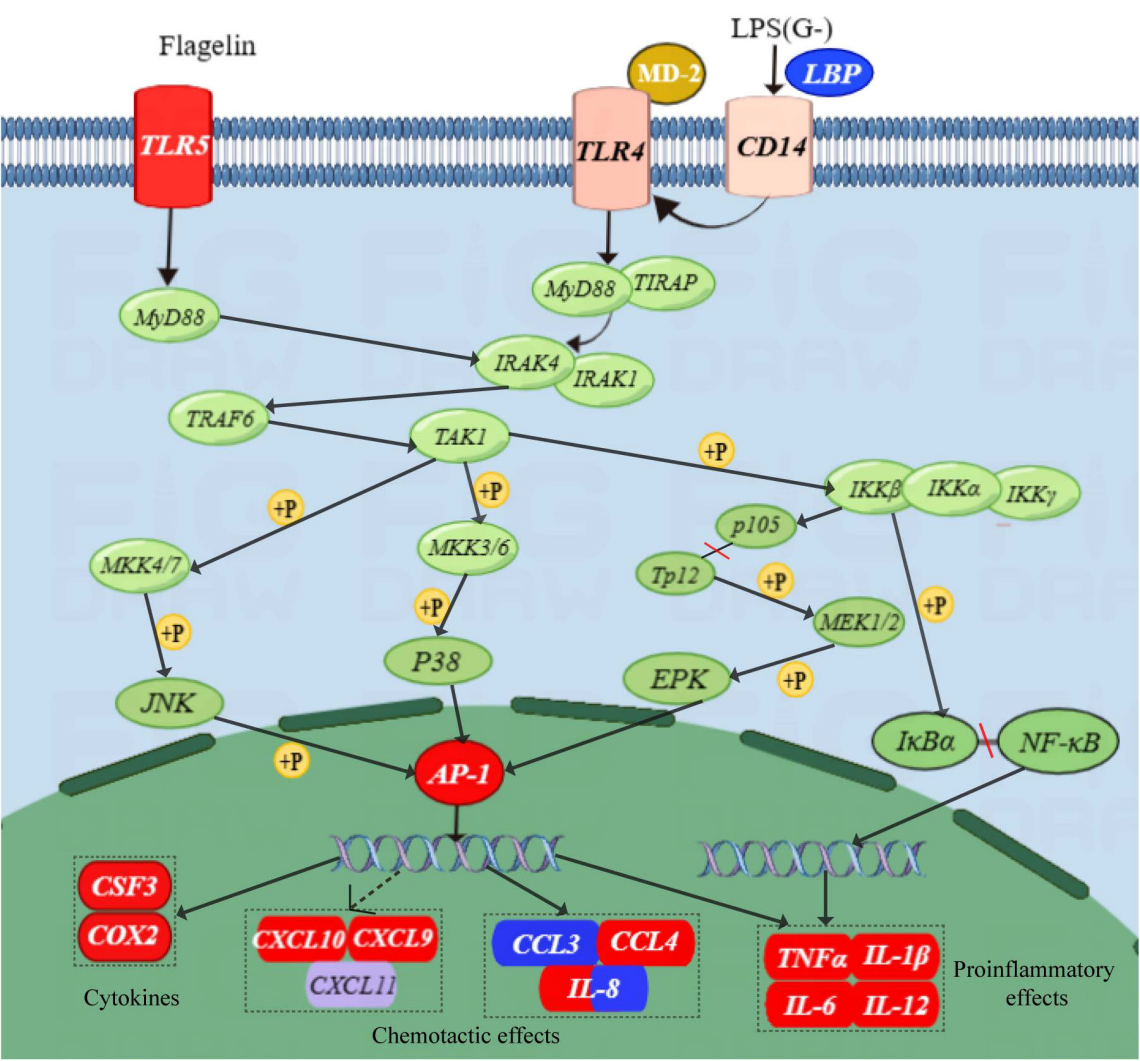
Schematic of TLR signaling pathway. Red indicates upward adjustment, blue indicates downward adjustment, and other colors indicate no significant change. Solid arrows indicate direct action, dotted arrows indicate indirect action; +P means phosphorylation.

**Figure 6** provides a map of the TLR signaling pathway based on the transcriptome results. Compared to the NZBD9-infected group, TLR5 receptor expression was up-regulated in the Δ*fliL*-infected group. Through NF-κB pathway and activation of nuclear transcription factor AP-1 and its family members, chemokines, proinflammatory cytokines and other cytokines are induced to express up-regulated, thus triggering inflammatory reaction and resisting bacterial infection.

### 3.5 Immune response of *P. plecoglossicida* NZBD9 and Δ*fliL* strains to hybrid grouper spleen

**Figure 7:**
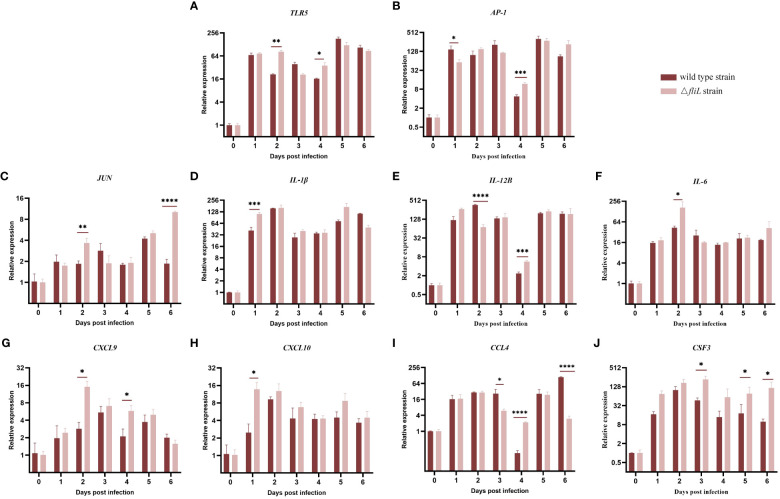
Relative expression levels of splenic immune-related genes in groupers infected with NZBD9 or Δ*fliL* strains from 0 to 6 dpi. **(A)** *TLR5* (Toll like receptor 5), **(B)** *AP-1* (AP-1 transcription factor), **(C)** *JUN* (AP-1 transcription factor subunit), **(D)** *IL-1β* (interleukin-1 beta), **(E)** *IL-12B* (interleukin-12 subunit beta), **(F)** *IL-6* (interleukin-6-like), **(G)** *CXCL9* (C-X-C motif chemokine 9-like), **(H)** *CXCL10* (C-X-C motif chemokine 10-like), **(I)** *CCL4* (C-C chemokine ligand 4), and **(J)** *CSF3* (colony stimulating factor 3). All data are expressed as mean ± SD, n = 3. Significant differences between two infection groups are indicated by an asterisk. ^*^ *P* < 0.05, ^**^ *P* < 0.01, ^***^ *P* < 0.001, ^****^ *P* < 0.0001.

To compare differences in immune response mechanisms within the spleen of hybrid groupers infected by the NZBD9 or Δ*fliL* strains from 0 to 6 dpi, the mRNA expression levels of 10 immune-related genes (*TLR5*, *AP-1*, *JUN*, *IL-1β*, *IL-12B*, *IL-6*, *CXCL9*, *CXCL10*, *CCL4*, and *CSF3*) were detected by qRT-PCR. Collectively, both the NZBD9 and Δ*fliL*-infected groups showed a substantial up-regulation in immune-related genes in the days following infection (1–6 dpi) compared to baseline (0 dpi) (**Figure 7**). Notably, the pattern recognition receptor-related gene *TLR5* was significantly up-regulated (*P* < 0.05) at 2 and 4 dpi in the Δ*fliL*-infected group compared to the NZBD9-infected group. The transcription factor-related gene *AP-1* and its family member gene *JUN* were significantly up-regulated at 1 and 4 dpi and at 2 and 6 dpi, respectively. Proinflammatory cytokine-related genes *IL-1β*, *IL-12B*, and *IL-6* also showed varying levels of up-regulation. Chemokine-related genes *CXCL9* and *CXCL10* were significantly up-regulated in the early phase of infection, while *CCL4* was significantly up-regulated in the late phase of infection. In addition, *CSF3*, related to immune cell activity regulation, was significantly up-regulated in the late stage of infection.

## 4 Discussion

The infection mechanisms utilized by pathogens are extremely complex, with flagella serving as key virulence factors that facilitate the entrance and adhesion of pathogens to host tissues (34, 35). Activation of the innate immune response in the host is triggered when its cell surface pattern recognition receptors identify flagellin, enabling the host to promptly respond and neutralize the invading pathogen (36). Recent studies have shown that flagellar genes, including *flgK* and *flgC*, in *P. plecoglossicida*—the primary pathogen responsible for visceral white spot disease in large yellow croakers and orange-spotted groupers—significantly impact its pathogenicity and are crucial for its infection process in orange-spotted groupers (32, 37). While the *fliL* gene is implicated in power generation of bacterial flagellar motility (38), the precise mechanism by which it affects the ability of *P. plecoglossicida* to infect hybrid grouper fish remains unclear.

This study used the Δ*fliL* strain to investigate the impact of the *fliL* gene from *P. plecoglossicida* on the pathogenicity toward hybrid groupers and their immune response mechanisms. Cumulative survival analysis revealed that hybrid groupers infected with the Δ*fliL* strain exhibited a 50% higher survival rate compared to those infected with the wild-type strain, suggesting a reduction in the pathogenicity of *P. plecoglossicida* following deletion of the *fliL* gene. Interestingly, silencing the flagellar gene *fliG* similarly attenuates *P. plecoglossicida* virulence in orange-spotted groupers (39). A hallmark of visceral white spot disease caused by *P. plecoglossicida* is the presence of white nodules within the fish spleen, which can engulf the entire organ in advanced stages (5, 6). Groupers infected with the Δ*fliL* strain showed significantly fewer white nodules in their spleens than those infected with the wild-type strain, suggesting that the *fliL* gene attenuates the symptoms of *P. plecoglossicida*-induced visceral white spot disease, consistent with the survival rate results. Host pathogen load can also reflect the extent of infection (40). Our results showed that bacterial load in vital immune organs, such as the spleen, head kidney, liver, and intestine, was consistently lower in fish infected with the Δ*fliL* strain compared to the wild-type strain, further underscoring the importance of the *fliL* gene in *P. plecoglossicida* infection of hybrid groupers.

The intestine, spleen, liver, and kidney, crucial for defending against pathogenic bacterial invasions, are key immune-related organs in fish and frequent targets of pathogenic attacks (41–43). For example, barramundi infected with *P. plecoglossicida* demonstrate granuloma formation in the spleen, hepatocellular swelling, steatosis necrosis, and glomerular collapse (44), while zebrafish show pathological shedding of intestinal villi, vacuolization of hepatocytes, and proliferation of splenic lymphocytes upon infection (45). In the present study, pathological analysis of infected hybrid groupers identified visible granulomas and many MMCs in the spleen in response to *P. plecoglossicida* infection. Granulomas serve as a protective mechanism by which immune cells organize the host to isolate and destroy infectious agents under continuous stimulation (46). As indicators of the host immune response, MMCs participate in intracellular bacterial deposition, iron ion storage, antigen capture and presentation, and degradation product collection (47–49). Similarly, the fish head kidney, rich in lymphocytes, plays an important role in pathogen phagocytosis and MMC-based immune memory (50). As a chemical, biological, physical, and immune barrier, the fish intestine plays a critical role in resisting pathogenic bacteria (51), while hepatic macrophages phagocytose pathogens that cross the intestinal barrier into the liver (43). In the current study, the tissue damage observed in the spleen, head kidney, liver, and intestine of hybrid groupers caused by the Δ*fliL* strain was less severe than that of the wild-type strain, suggesting that the Δ*fliL* strain is much less pathogenic and hybrid groupers can mobilize their immune organs for effective eradication.

RNA-seq technology has been widely used to study the mechanisms of pathogen invasion and host immune responses (52–54). In this study, RNA-seq was employed to further understand the mechanism underlying the effects of the *fliL* gene on infection of hybrid groupers and their resulting immune response. Among the 2 238 DEGs identified in the transcriptome, 1 112 were up-regulated and 1 126 were down-regulated. These DEGs were predominantly enriched in immune system-related pathways and signal transduction pathways, including the TLR signaling pathway, cytokine-cytokine receptor interaction, complement and coagulation cascade reaction, and IL-17 signaling pathway. Immune defense is energetically demanding, requiring metabolic adjustments by the host to redistribute energy resources intended for growth and development to sustain an effective immune response (55). Pathogenic exposure triggers the activation of many processes related to lipid, carbohydrate, and amino acid metabolism, marshaling these competent molecules to support the immune response (56–58). Consistently, our results showed enrichment in metabolism-related pathways, including retinol and arachidonic acid metabolism, in many up-regulated DEGs, suggesting an effective immune response and increased energy expenditure by the host.

In contrast to mammals, the innate immune system in fish assumes a more important role in combating pathogenic infections (59–61), with pattern recognition receptors (PRRs) playing a critical role in recognizing microbial pathogens and initiating innate immune responses (62). TLRs are a type of cell surface PRR with the ability to specifically recognize ligands, mobilize multiple transcription factors (e.g., NF-κB, AP1, and IRFs), and activate intracellular innate immunity (63, 64). Notably, TLR5, known for its specific recognition of bacterial flagellin, induces an immune response and plays an important role in protecting fish from disease (65). Activation of TLR5 stimulates the expression of proinflammatory cytokines via the Myd88 and NF-κB pathways (62, 66), as well as transcription factor AP-1, which, in turn, induces the production of chemokines and other cytokines (66, 67). As a core AP-1 transcription factor, JUN is also involved in the inflammatory response, ensuring proper immune system activation and immune cell proliferation and differentiation (68). The chemokine superfamily, comprising chemotactic cytokines, is essential for directing immune cells to the site of infection, thereby facilitating an immune response (69).

In this study, *AP-1* and its family gene *JUN* were significantly up-regulated in the spleen of infected groupers, as were proinflammatory cytokine-related genes *IL-1β*, *IL-12B*, and *IL-6* to varying degrees. IL-1β, a critical proinflammatory factor produced by macrophages and other immune cells, plays a major role in stimulating and mediating inflammation (70). Our results also showed that chemokine-related genes *CXCL9*, *CXCL10*, and *CCL4* were also up-regulated, indicating potential involvement in recruiting inflammatory cells (e.g., neutrophils, T-lymphocytes, and natural killer cells) to the site of inflammation to modulate the immune response (71–73). Of note, *CXCL10* is crucial for controlling the inflammatory response and maintaining the balance between tissues and stroma (74). The *CSF3* gene was also significantly up-regulated in the middle and late stages of infection. Granulocyte colony-stimulating factor (G-CSF or CSF3) stimulates granulocyte production and regulates neutrophil migration and antimicrobial activity (75). In conclusion, deletion of the *fliL* gene induced changes in multiple immune-related and signal transduction pathways in the host, enabling groupers to initiate innate immunity-driven defense through an inflammatory response involving a wide range of cytokines.

## 5 Conclusions

The results of this study underscore the critical role of the *fliL* gene in the pathogenicity of *P. plecoglossicida*. Deletion of the *fliL* gene increased the survival of hybrid groupers infected with *P. plecoglossicida*, reduced bacterial load in the spleen, head kidney, liver, and intestine, and mitigated tissue damage. Transcriptomic analysis revealed that Δ*fliL* strain infection in hybrid groupers activated the TLR5-mediated NF-κB pathway and transcription factor AP-1 in the MyD88 pathway, resulting in an effective immune response. Up-regulation of multiple genes associated with inflammatory response in the spleen further suggested a critical role for the *fliL* gene in the activity of multiple cytokines and in regulating the immune response to *P. plecoglossicida* in hybrid groupers.

## Data availability statement

The RNA-seq data were deposited in the NCBI GenBank SRA database under accession numbers SRP432909 (NZBD9 strain group) and SRP433934 (ΔfliL strain group).

## Ethics statement

The animal study was approved by the Ethics Committee of Jimei University (license No. JMULAC201159). The study was conducted in accordance with the local legislation and institutional requirements.

## Author contributions

LS: Investigation, Writing – original draft. LZ: Investigation, Writing – original draft. QL: Investigation, Methodology, Writing – original draft. LH: Methodology, Resources, Writing – original draft. YQ: Conceptualization, Resources, Writing – original draft. ZZ: Conceptualization, Resources, Writing – review & editing. XW: Conceptualization, Resources, Writing – review & editing. HH: Data curation, Methodology, Writing – original draft. JNZ: Methodology, Resources, Writing – original draft. JLZ: Conceptualization, Resources, Writing – original draft. QY: Funding acquisition, Supervision, Writing – review & editing.
