# Supplementary material for: Role of the Pseudomonas plecoglossicida fliL gene in immune response of infected hybrid groupers (Epinephelus fuscoguttatus ♀ × Epinephelus lanceolatus ♂)
Source: Front Immunol. 2024 Jul 4;15:1415744. doi: 10.3389/fimmu.2024.1415744 (PMC11254626; doi:10.3389/fimmu.2024.1415744)
Supplement: Supplementary file 1 [file DataSheet_1.doc]

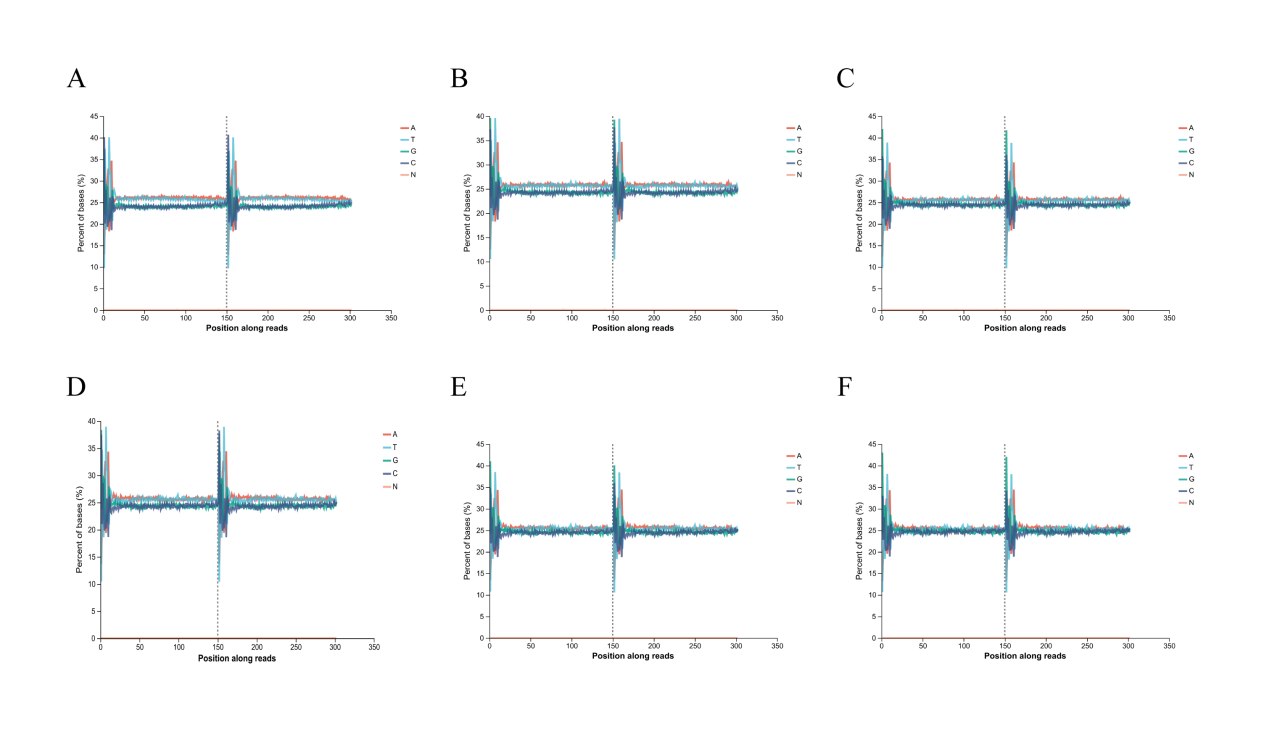
**Supplementary Fig. 1**: Bases content along clean reads. The horizontal coordinate is the base coordinate of reads, and the vertical coordinate is the percentage of base A, C, G, T and N of all reads at the sequencing location, where N refers to the fuzzy base. Different bases are represented in different colors: A is red ; T is blue; G is green; C is dark blue; N is orange. (A), (B), (C) are Δ*fliL* strain-infected group, and (D), (E), (F) are NZBD9 strain-infected group.
