# Supplementary material for: Role of the Pseudomonas plecoglossicida fliL gene in immune response of infected hybrid groupers (Epinephelus fuscoguttatus ♀ × Epinephelus lanceolatus ♂)
Source: Front Immunol. 2024 Jul 4;15:1415744. doi: 10.3389/fimmu.2024.1415744 (PMC11254626; doi:10.3389/fimmu.2024.1415744)
Supplement: Supplementary file 2 [file DataSheet_2.doc]

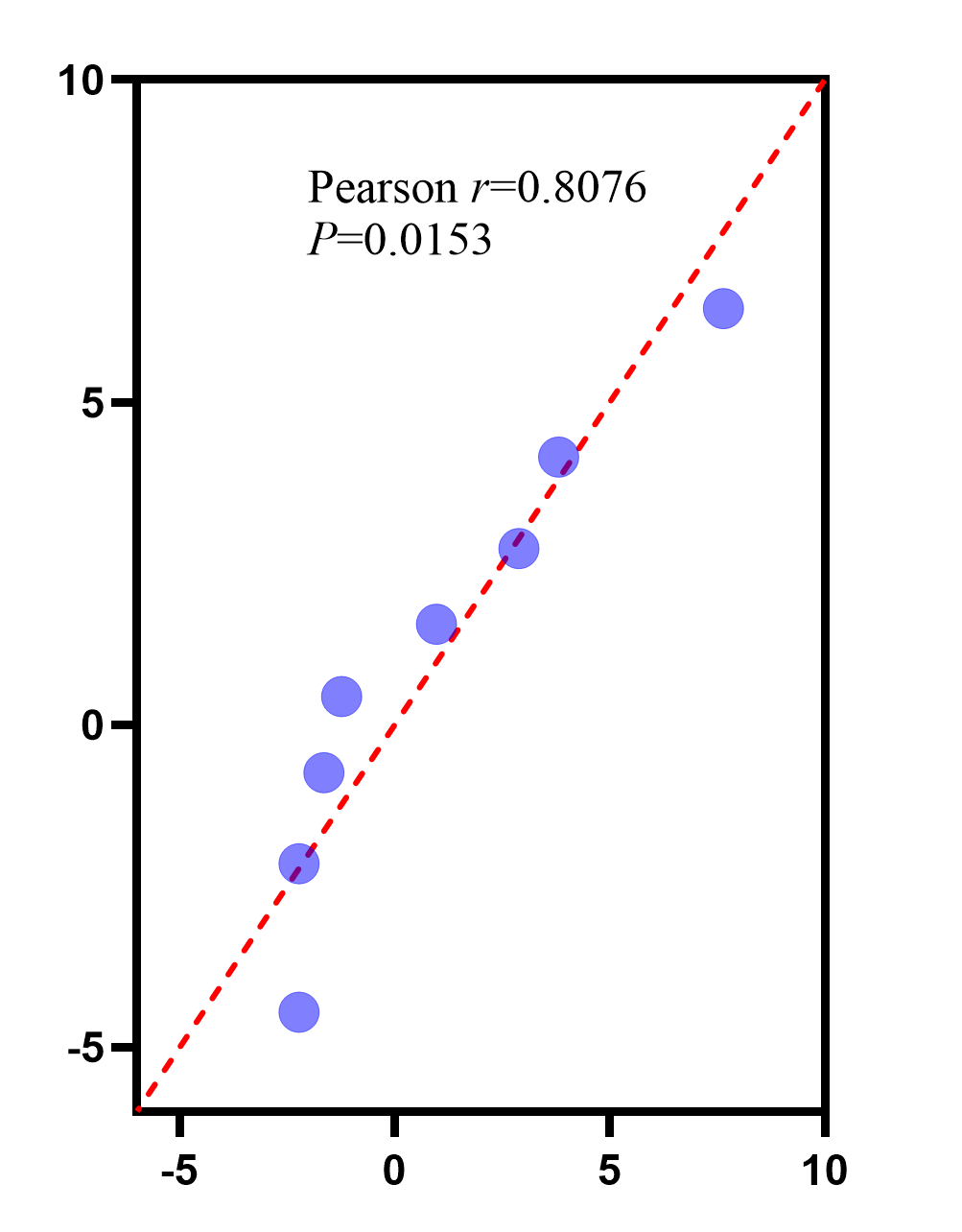


**Supplementary Fig. 2**: Correlation analysis between qRT-PCR results and RNA-seq. r is the correlation coefficient, and *P* ＜0.05 indicates a significant correlation.
