# Supplementary material for: Role of the Pseudomonas plecoglossicida fliL gene in immune response of infected hybrid groupers (Epinephelus fuscoguttatus ♀ × Epinephelus lanceolatus ♂)
Source: Front Immunol. 2024 Jul 4;15:1415744. doi: 10.3389/fimmu.2024.1415744 (PMC11254626; doi:10.3389/fimmu.2024.1415744)
Supplement: Supplementary file 3 [file Table_1.doc]

**Supplementary Table 1**. The sequence of primers for qRT-PCR

| Primer | Sequence (5’-3’) |
| --- | --- |
| gyrB-F | TGCTGAAGGACGAGCGTTCG |
| gyrB-R | GAATATTGTTGGTGAAGCAGGCTA |
| β-actin-F | GGCTACTCCTTCACCACCACAG |
| β-actin-R | TCTGGGCAACGGAACCTCT |
| TLR5-F | TCTCTGGTGAGCAAGATGTGGATG |
| TLR5-R | CGATGAGGCATGATGGGAAACAAC |
| AP-1-F | GAAGGATTTGCTGATGGCTTTGTC |
| AP-1-R | ACGGCGGAGGATGAAGATGG |
| JUN-F | GAAGGATTTGCTGATGGCTTTGTC |
| JUN-R | ACGGCGGAGGATGAAGATGG |
| IL-1β-F | AACTGTGGCTCTGGGCATCAAG |
| IL-1β-R | AGGCTGTCTTTGGTAATCGTCTCC |
| IL-6-F | CGACAACCCCAGCACCTTCC |
| IL-6-R | GCCATAGTCCTGACAGCCAGAC |
| IL-12B-F | CACAGCCTTCGCACCTCTCC |
| IL-12B-R | CGTAGCACAGTCCAGCACATAGG |
| CXCL9-F | GTGAGAGAGTCAGACAGCACG |
| CXCL9-R | CTCTTTGGGTAGACTGCGAGG |
| CXCL10-F | TGTGAACGCGTCAGGAACAT |
| CXCL10-R | TTGGGGTTCAGGCAGTAAGG |
| CCL4-F | ATGAGAGCGAGGTGCCAAGA |
| CCL4-R | CCAGGTAGGTGATGAGCGTCTTC |
| CSF3-F | TGGCATCCACCATTGGCATCC |
| CSF3-R | CCAGCCGAGGAGAGACAGAAATC |
