# Supplementary material for: Role of the Pseudomonas plecoglossicida fliL gene in immune response of infected hybrid groupers (Epinephelus fuscoguttatus ♀ × Epinephelus lanceolatus ♂)
Source: Front Immunol. 2024 Jul 4;15:1415744. doi: 10.3389/fimmu.2024.1415744 (PMC11254626; doi:10.3389/fimmu.2024.1415744)
Supplement: Supplementary file 4 [file Table_2.doc]

**Supplementary Table 2**. Software and database information

| Soft/Database | Version | Analysis | Source |
| --- | --- | --- | --- |
| fastp | Version 0.19.5 | Sequencing data quality control | https://github.com/OpenGene/fastp |
| Trinity | Version v2.8.5 | Assembly | https://github.com/trinityrnaseq/trinityrnaseq |
| TransRate | Version v1.0.3 | Assembly | http://hibberdlab.com/transrate/index.html |
| CD-HIT | Version v4.5.7 | Assembly | https://github.com/weizhongli/cdhit |
| BUSCO | Version 3.0.2 | Assembly | https://busco.ezlab.org/ |
| RSEM | Version 1.3.1 | Expression level analysis | http://deweylab.biostat.wisc.edu/rsem/ |
| DESeq2 | Version 1.24.0 | Differentially expressed genes analysis | http://bioconductor.org/packages/stats/bioc/DESeq2/ |
| Goatools | Version 0.6.5 | GO enrichment analysis | https://files.pythonhosted.org/packages/bb/7b/0c76e3511a79879606672e0741095a891dfb98cd63b1530ed8c51d406cda/goatools- |
| KOBAS database | Version 3.0 | KEGG enrichment analysis | http://kobas.cbi.pku.edu.cn/ |
