# Supplementary material for: Role of the Pseudomonas plecoglossicida fliL gene in immune response of infected hybrid groupers (Epinephelus fuscoguttatus ♀ × Epinephelus lanceolatus ♂)
Source: Front Immunol. 2024 Jul 4;15:1415744. doi: 10.3389/fimmu.2024.1415744 (PMC11254626; doi:10.3389/fimmu.2024.1415744)
Supplement: Supplementary file 5 [file Table_3.doc]

**Supplementary Table 3**. Primer sequence for transcriptome validation

| Primer | Sequence (5’-3’) |
| --- | --- |
| IRF1-F | CAATGAACTCACTGCCTGACATCG |
| IRF1-R | TCTTTGCCTCCTTTGCTTTGCTTC |
| IL4I1-F | GAAGTGACTGGTGGGTCTGATCTC |
| IL4I1-R | CTGGTGGTCTGTCTGGTACGATAC |
| IL27B-F | ATCTGAACGGTTGTGGCACT |
| IL27B-R | CAGCAGGTTTCTGACGTCCT |
| SOX7S-F | CCACCACCTCCACCATCATAACC |
| SOX7S-R | GCACCAACACTCATTCCTGAACTC |
| CCR7-F | CGCTACTTCGTCATCGCCAA |
| CCR7-R | CATTGCTGGAGTACGGGGTG |
| C3-F | GCTCAACCAATACGCCACAAG |
| C3-R | CTGGGATAAGCTGCACTGACA |
| CMPK2-F | CGGAGGCCAGAAGTAAACCA |
| CMPK2-R | GAAGGCCCTGCGGATAAGTG |
| CXCR3-F | CTGGGCCTGAGTGTAGTGGA |
| CXCR3-R | GAGACCTGAAGATGCGGATGT |
