# Supplementary material for: Role of the Pseudomonas plecoglossicida fliL gene in immune response of infected hybrid groupers (Epinephelus fuscoguttatus ♀ × Epinephelus lanceolatus ♂)
Source: Front Immunol. 2024 Jul 4;15:1415744. doi: 10.3389/fimmu.2024.1415744 (PMC11254626; doi:10.3389/fimmu.2024.1415744)
Supplement: Supplementary file 6 [file Table_4.doc]

**Supplementary Table 4**. RNA-seq data statistics

| Sample | Clean reads | Clean bases | Error rate(%) | Q20(%) | Q30(%) | GC content(%) |
| --- | --- | --- | --- | --- | --- | --- |
| NZBD9 strain-infected group1 | 49133136 | 7192623854 | 0.0256 | 97.86 | 93.65 | 48.87 |
| NZBD9 strain-infected group2 | 56698316 | 8329274571 | 0.0257 | 97.8 | 93.53 | 49.24 |
| NZBD9 strain-infected group3 | 57584224 | 8472544676 | 0.0256 | 97.85 | 93.6 | 49.51 |
| Δ*fliL* strain-infected group1 | 71905156 | 9164309722 | 0.025 | 98.17 | 94.59 | 48.27 |
| Δ*fliL* strain-infected group2 | 62736720 | 9006122990 | 0.0253 | 97.95 | 93.97 | 48.7 |
| Δ*fliL* strain-infected group3 | 43348828 | 6421290471 | 0.0253 | 97.94 | 93.87 | 49.06 |
